# The Role of Prenatal Microglial Activation and Its Sex Differences in the Development of Neuropsychiatric Disorders and Neurodegenerative Diseases

**DOI:** 10.3390/ijms26189250

**Published:** 2025-09-22

**Authors:** Alexander Sergeevich Lyamtsev, Alexandra Vladislavovna Sentyabreva, Anna Mikhailovna Kosyreva

**Affiliations:** 1 Avtsyn Research Institute of Human Morphology of Petrovsky National Research Centre of Surgery, 117418 Moscow, Russiaalexandraasentyabreva@gmail.com (A.V.S.); 2 Research Institute of Molecular and Cellular Medicine, Peoples’ Friendship University of Russia (RUDN University), 117198 Moscow, Russia

**Keywords:** microglia, maternal immune activation, autistic spectrum disorder, neuropsychiatric disorders, neurodegeneration, Alzheimer’s disease, aging, inflammaging

## Abstract

Maternal Immune Activation (MIA) is a phenomenon of pathophysiological stimulation of the maternal immune system during gestation which potentially leads to functional and structural disturbances of fetal neurogenesis. It occurs due to the alteration of paracrine signals between the maternal organism and the developing nervous system of the fetus. Any disturbances in the brain at embryonic and early postnatal stages might compromise its natural developmental trajectory, which could potentially increase the risk of developing neuropsychiatric disorders, such as schizophrenia, autistic spectrum disorder (ASD), attention deficit hyperactivity disorder (ADHD), major depressive and bipolar disorders, etc. Presumably, all these conditions could initiate the development of age-related cognitive impairment in late ontogenesis, including Alzheimer’s disease (AD), Parkinson’s disease (PD), and others. As the main immune cell population in the CNS, microglia both mediate its proper development and receive pathological stimuli from the maternal organism. This could lead to microglia premature activation and could become a part of the mechanisms of the fetal CNS development alterations. In this review, we discuss the role of prenatal activation of microglia in neuropsychiatric disorders and neurodegenerative disease development. We highlight approaches to modeling MIA, as well as sex differences in the morphological and functional state of microglia in the context of physiological conditions. There is a hypothesis discussed regarding the contribution of these distinctions to neuropsychiatric disorders and neurodegenerative disease incidence, prevalence, and progression in males and females.

## 1. Introduction

Environmental and maternal pathological conditions during pregnancy have a significant influence on fetal immune and nervous system development [1]. Their inauspicious impact greatly increases the risk of developing neuropsychiatric disorders (NPDs) and neurodegenerative diseases [2]. Maternal Immune Activation (MIA) hypothesis postulates a pathogenic mechanism leading to excessive stimulation of the maternal immune system during gestation [3]. Potentially, it could induce functional and structural disturbances of fetal neurogenesis [4]. This phenomenon’s mechanism consists of the alteration of paracrine signals between the maternal organism and the fetus’ developing nervous system [4]. As a result, the risk of disruptions in the regulation of neuroplasticity, differentiation, and histoarchitecture of the central nervous system (CNS) might be increased significantly [4]. The adverse impact of MIA was mostly confirmed in experimental studies involving rodents and non-human primates. Also, this paradigm is strongly supported by epidemiological and experimental data due to a correlation between maternal immunological responses and subsequent disturbances in the offspring’s CNS development. Such disruptions include changes in pro-inflammatory cytokines’ expression and secretion and microglial phenotypes shifts during critical periods of embryogenesis [5]. The prenatal period is characterized by extreme vulnerability of neuronal differentiation and plasticity processes, since the fundamental mechanisms of embryo- and neurogenesis are most active at this stage. The key events of this period are (1) the neural precursors proliferation, (2) migration of neurons and glial cells to different brain regions, (3) region-specific phenotypes differentiation, (4) formation of axons and dendritic structures, and (5) synaptic contact establishment [6]. The process of excessive structural elements elimination occurs simultaneously. It includes the removal of synapses, dendritic spines, and fragments of axons and dendrites to ensure the neural networks’ optimization. The final stage is the myelination of axons by oligodendrocytes, which is vital for the effective nerve impulses transmission. All these processes require strict temporal and spatial coordination and are quite sensitive to various endogenous and exogenous factors capable of inducing pathological deviations. The critical embryonic periods are the end of the second and the beginning of the third trimester of pregnancy. Disturbances in the developing brain at these stages might change its natural developmental trajectory, which increases the risk of developing neuropsychiatric disorders (NPDs), such as schizophrenia, autistic spectrum disorder (ASD), attention deficit hyperactivity disorder (ADHD), major depressive and bipolar disorders, etc. All these conditions are potentially able to initiate the development of age-related cognitive impairment in late ontogenesis, including Alzheimer’s disease (AD), Parkinson’s disease (PD), and other neurodegenerative diseases [1,2,4,5,6,7,8,9].

Microglia, as the resident mononuclear phagocytes of the CNS, play the key roles in embryonic neurogenesis. In particular, they take an active part in the processes of proliferation, differentiation of neural progenitor cells (NPCs) into neuroblasts, and maintaining their survival, migration, maturation, and integration into the neural network. Microglia participate in interneuron connections formation via pruning weak or less active synapses and eliminate excessive neurons without any functional connections. These cells also participate in gliogenesis via promoting differentiation and proliferation of astrocytes and oligodendrocytes, which was demonstrated in vitro [10,11]. Various adverse stimuli and pathological conditions might make microglia become activated and shift to the pro-inflammatory (cytotoxic) phenotype. Such cells secrete a large spectrum of inflammation mediators, which subsequently could have a negative impact on the processes of normal neurogenesis and potentially increase the risk of NPDs and neurodegenerative diseases developing.

The association between MIA and neuropsychiatric disorders is actively studied [2,12,13,14], and the role of prenatal microglial activation in the pathogenesis of neurodegenerative diseases in offspring remains a research frontier. This review summarizes experimental and clinical data concerning the role of microglia in the pathogenesis of NPDs and neurodegenerative diseases in offspring. Here, we discuss the mechanisms of prenatal microglial activation and the ways in which it contributes to the development of neurological disorders in offspring, as well as evaluating potential molecular and cellular targets for therapeutic intervention.

## 2. Literature Search Strategy

To ensure transparency and comprehensiveness, we conducted a structured literature search using databases such as PubMed, Scopus, and Web of Science. The search covered publications from January 2000 to July 2025 and included combinations of the following terms: “MIA microglia”, “MIA neuropsychiatric”, “neurodevelopmental diseases microglia”, “MIA ASD ADHD”, and “maternal immune activation neurodevelopment.” Inclusion criteria comprised peer-reviewed original research and reviews focusing on human and rodent models of MIA, microglial activation, and neurodevelopmental outcomes. Studies were excluded if they lacked relevance to neuro-immune mechanisms or did not report sex-specific or timing-dependent effects.

## 3. Maternal Immune Activation (MIA)

In the 1980s, researcher David Barker performed epidemiological studies in the UK and revealed a negative correlation between birth weight and adult mortality rates from cardiovascular diseases, including coronary heart disease [14]. Based on these results, it was suggested that prenatal and early postnatal ontogenesis are critical periods for the subsequent normal development of the organism and the nervous system in particular. Later, David Barker’s hypothesis was transformed into the ontogenetic concept of health and disease formation (Developmental Origins of Health and Disease—DOHaD). This phenomenon is based on the relationship between the impact of environmental factors on pregnant women and their offspring’s health throughout their life [15]. In 1988, Mednick S. et al. published a seminal paper that displayed a link between maternal influenza virus infection during pregnancy and the subsequent development of schizophrenia in the offspring. Over the decades, numerous epidemiological studies have confirmed the link between viral infections in the mother and the risk of developing nervous system pathology in offspring, particularly schizophrenia and ASD [9,15,16,17,18,19,20,21,22,23,24,25,26,27,28,29,30].

MIA is one of the DOHaD phenomena. It occurs when maternal infections, autoimmune and allergic diseases, atherosclerosis, malignant neoplasms, hyperhomocysteinemia, excess body weight, stress, or alcohol consumption activate the mother’s immune response [15]. This state could potentially markedly increase the risk of fetal developmental disorders, including disorders of the CNS [30,31,32].

### 3.1. Epidemiological Data Regarding the Role of MIA in the Development of Neuropsychiatric Disorders

The results of clinical and epidemiological studies confirm the existence of a link between gestational infection and an increased risk of developing CNS disorders [1,22]. Recent data display a correlation between an increased incidence of ASD and schizophrenia in children and mothers’ gestational infections, such as rubella, influenza, measles, mumps, and poliomyelitis. Many pathogens can infect the fetus directly by transplacental transmission, as well as during delivery or breastfeeding. The most common ones are chickenpox virus, hepatitis B and C virus, chlamydia, gonococci, listeria, HIV, enteroviruses, Zika virus, and TORCH complex infections (Toxoplasmosis, other (including syphilis, varicella, and other infections), Rubella, Cytomegalovirus, and Herpes simplex virus). This leads to unfavorable pregnancy outcomes, such as low birth weight, preterm birth, or even stillbirth [31,32]. At the same time, any prevalent infections might increase the risk of neuropsychiatric disorders in offspring as well, despite the absence of their transplacental transmission to the fetus. A meta-analysis including data from 15 studies demonstrated that common maternal infections occurring in pregnancy, such as pyelonephritis and cystitis caused by E. coli and streptococcus, Lyme borreliosis, and dermatitis caused by skin mites, are associated with a 13% increased risk of ASD in the offspring [23,33]. According to other data, maternal genitourinary infections during pregnancy increase the risk of ADHD in the offspring by 26–33%, while viral respiratory infections cause a 3-fold increase [34,35,36]. Mothers’ autoimmune diseases could also be a risk factor for ASD and ADHD development [37,38,39,40]. A correlation between bronchial asthma in the mother and a higher risk of ASD and ADHD developing in the offspring has been demonstrated in several population studies [41,42,43,44].

Infectious and autoimmune diseases during pregnancy are able to induce systemic inflammatory responses activation through multiple pathogenic mechanisms, including the release of pro-inflammatory cytokines, chemokines, and inflammatory mediators. At the same time, exposure to environmental factors (e.g., air pollution, toxic substances), psychosocial stressors (chronic stress, anxiety, and depressive disorders), and metabolic disturbances can prevent effective resolution of the acute inflammatory response, promoting the transition to a state of persistent chronic systemic low-grade inflammatory background. This condition is characterized by persistent activation of immune cells and maintenance of an elevated baseline level of circulating pro-inflammatory markers. It potentially has long-term effects on the developing fetus, including disturbances in neuron–glia interactions and epigenetic regulation of gene expression. There is a growing pool of evidence that chronic inflammatory conditions during pregnancy are great risk factors for the development of NPDs in the offspring [1,45,46]. Maternal overweight was associated with an increased risk of ASD and ADHD in the offspring as well [47,48]. In addition, mothers with a low socioeconomic status are twice as likely to have a child with ADHD or ASD than mothers with an average or high socioeconomic status [49,50].

The period of pregnancy, when the impact of negative factors occurs, also affects the development of NPDs in the offspring. Studies have shown that there is a correlation between pregnant women’s asthma in the first and second trimesters of pregnancy (but not in the third one) and the risk of ASD developing in the offspring. Maternal bacterial infections in the third trimester increased the possibility of children developing ASD and ADHD [23,51]. Recent comparative studies have provided convincing evidence that systemic pro-inflammatory activation of maternal immunity during pregnancy is a significant risk factor for the development of NPDs in the offspring [9,52,53]. However, the mechanisms of this phenomenon could vary significantly depending on the etiology of the primary pathological condition.

Autoimmune diseases and asthma have a direct immune response leading to increased levels of pro-inflammatory cytokines TNF-α, IL-6, and IL-1β, which induce microglial activation and compromise neuroplasticity processes in the fetus. Experimental models demonstrate that MIA induces the expression of upregulation of C1q and C3 and their receptors’ genes on microglia. This imbalance in the complement systems leads to impairment of synaptic elimination [54,55]. Recent studies have highlighted the role of IL-17a as both a key cytokine of autoimmune reactions and a mediator involved in the development of NPDs via disruptions of hippocampal functions and behavioral responses in the offspring [56,57].

Obesity has a more complex impact on fetal neurodevelopment via a chronic low-grade inflammatory background accompanied by increased secretion of adipokines, such as leptin and adiponectin. These hormones are capable of crossing the blood–brain barrier (BBB) and influencing neurogenesis through metabolic pathways alterations and oxidative stress response regulation [58,59]. Studies also revealed a correlation between maternal obesity and impaired axonal organization, possibly due to decreased levels of Brain-Derived Neurotrophic Factor (*BDNF*) mRNA and mature functionally active protein, as well as other neurotrophic factors [60].

Chronic mental stress activates the hypothalamic–pituitary–adrenal (HPA) axis, resulting in a systemic increase in corticosterone level. It modulates the function of NMDA-(N-methyl-D-aspartate) and AMPA (α-amino-3-hydroxy-5-methyl-4-isoxazolepropionic acid) receptors, especially NR2B subunits, through post-translational upregulation of phosphorylation mechanisms. These changes disrupt synaptic plasticity, reduce the level of long-term potentiation (LTP), and increase long-term depression (LTD), which is associated with the development of cognitive deficits and behavioral dysfunction. There are studies confirming that chronic stress might cause epigenetic changes, particularly hypomethylation of genes associated with the nervous system development, such as *BDNF* and *NR3C1* (glucocorticoid receptor) [61,62]. This can result in the disruption of proper neuronal migration and synapse formation.

There are some sex differences in the development of stress reactions. In females, both acute and chronic stress causes a more pronounced and prolonged release of corticosteroids. It is associated with estrogen’s effect on the hypothalamus and pituitary gland. Meanwhile, a more rapid formation of theHPA axis negative feedback is observed in males under stress conditions, contributing to quicker homeostasis restoration after stress [63]. Sex differences in the glutamate system regulation are known to contribute to the response to acute stress factors that affect cognitive functions. A study on C57BL/6J mice showed that 24 h immobilization stress leads to disruption of novel object location (NOL) in both sexes. However, novel object recognition (NOR) was disrupted only in males. It might be associated with sex and time differences in the expression level of NMDA and AMPA receptor subunits in the dorsal hippocampus. The NOR test showed increased expression of *Glun2b* (NMDA), decreased *Glua1* (AMPA), and an imbalance between them in males. In females, there was a moderate increase in Glua2 (AMPA), stable expression of *Glun1* and *Glun2a*, and no significant changes in *Glun2b*. After the NOL test, activation of NMDA receptors with predominance of *Glun2b* expression was observed in males, while females displayed adaptive changes in AMPA receptors [64]. Overall, these data suggest some sex-related mechanisms are involved in both regulation of reactions on stress conditions during pregnancy and the predisposition to increased vulnerability of the CNS to adverse impact in case of prenatal MIA exposure, depending on the sex of the offspring.

Data are summarized in Table 1.

As follows from Table 1, epidemiological data confirm that MIA and systemic pro-inflammatory background during pregnancy are pivotal risk factors for NPDs development in the offspring. Infections suffered during gestation have a direct correlation with an increased risk of such disorders. At the same time, autoimmune and allergy diseases, obesity, psychosocial stress, and exposure to inauspicious environmental factors contribute to the development of chronic low-grade inflammation, which has a long-term impact on the developing fetus. Although there was significant progress in understanding the role of MIA in NDPs and neurodegenerative diseases, most of the mechanisms of their development remain poorly understood. The relations between different types of immune stimuli, their exposure timing, and their specific effects on neurodevelopment require further investigation. In addition, more in-depth studies of epigenetic mechanisms, including DNA methylation, histone modification, and regulation of non-coding RNAs are needed to elucidate how these processes shape the long-term programming of neuronal connections.

### 3.2. Approaches to MIA Modeling

Both the innate and adaptive immune responses are activated in the presence of infectious or inflammatory processes in the mother’s body. They are accompanied by a complex change in the profile of circulating pro-inflammatory mediators. Key cells of the innate immune system include macrophages, monocytes, neutrophils, and dendritic cells. They start to secrete pro-inflammatory cytokines (IL-1β, IL-6, TNF-α), chemokines (CXCL8/IL-8, CCL2/MCP-1), and other signaling molecules, including the production of acute phase proteins and complementary factors. These biologically active substances can interact with the placenta through paracrine mechanisms, modulating its functioning and crossing the placental barrier.

Microglial activation occurring due to maternal cytokines impact may result in dysregulation of neuroplasticity processes such as neurogenesis, neuronal migration, cell differentiation, synapse formation, and elimination of excessive ones Studies on the effects of MIA exposure on fetal neurodevelopment in humans are limited to prenatal and postnatal periods, so these effects are mainly described in retrospective epidemiological studies. Hence, to establish specific pathophysiological mechanisms of MIA’s influence on brain development in the offspring, the use of animal models is necessary.

There are a number of MIA models which use various immune activators, including viral and bacterial components. The most widely used ones are Poly I:C, the Toll-Like Receptor 3 (TLR3) agonist, and LPS, the TLR4 agonist. In some studies, the TLR11/TLR12 agonist T. gondii [65], the selective TLR7 agonist imiquimod, and the TLR7/TLR8 agonist resiquimod [66,67] are used. The application of live pathogens such as influenza virus [68] in animal models could be conducted with significant biosafety precautions, which are not available in most academic and clinical research centers. Thus, experimental studies of MIA are predominantly carried out using viral and bacterial components, particularly Poly I:C and LPS.

#### 3.2.1. MIA and Poly I:C

Poly I:C (polyinosinic–polycytidylic acid) is a double-stranded RNA molecule and is a ligand for TLR3 due to its structural similarity to viral double-stranded RNA. TLR3 is expressed on neurons, astrocytes, and microglia in the human CNS. It is also expressed in fibroblasts and epithelial cells, including endothelial, ciliated, corneal, cervical, biliary, and intestinal cells [65]. TLR3 is predominantly localized to endosomes in innate and adaptive immunity cells, such as natural killer cells, macrophages, dendritic cells, and B-lymphocytes [66].

Poly I:C/TLR3 interaction triggers a complex cascade of immune and metabolic reactions that plays a key role in antiviral defense and inflammatory processes. The entry of Poly I:C into cellular endosomes occurs via the endocytosis mechanism, where it binds to TLR3 specifically recognizing the structure of double-stranded RNA. After binding, TLR3 assembles and provides a platform for the recruitment of the adapter protein TRIF (TIR domain-containing adapter-inducing interferon-β) or TICAM1. Some studies highlight the importance of TRIF as a central activation node of antiviral and inflammatory responses. It can modulate cellular metabolic pathways, such as glycolysis and oxidative phosphorylation, thereby contributing to the maintenance of energy homeostasis under inflammatory conditions [67]. TRIF initiates a cascade of reactions leading to nuclear transcription factor NF-κB activation. It involves the recruitment of TRAF (TNF receptor-associated factor) family proteins, in particular TRAF6, to activate the IKK complex (IκB kinase complex). Phosphorylation of IκBα, the key NF-κB inhibitor, leads to its degradation and NF-κB release. It initiates the transcription upregulation of pro-inflammatory cytokines such as IL-6, TNF-α, IL-1β, and CXCL3/10 chemokines genes. There are numerous studies showing that NF-κB is involved in the expression of microRNAs (miRNAs) regulation, which is one of the regulatory components of inflammatory processes [68,69,70]. TRIF also activates recruitment of TBK1 (TANK-binding kinase 1) and IKKε to phosphorylate the transcription factor IRF3 (Interferon Regulatory Factor 3). Activated IRF3 dimerizes and induces transcription of interferons IFN-β and IFN-α genes in cell nuclei. Although the main effect of TLR3 activation is pro-inflammatory cytokines secretion induction, some studies demonstrated the production of the anti-inflammatory cytokine IL-10. The secretion of IL-10 can be induced via alternative pathways, such as STAT3 (Signal Transducer and Activator of Transcription 3) activation [71].

In addition, TRIF activates MAPK (mitogen-activated protein kinase) signaling pathways, including JNK (c-Jun N-terminal kinases), p38 MAPK, and ERK (extracellular signal-regulated kinase). JNK is involved in apoptosis and inflammation regulation via enhancing pro-inflammatory gene expression. p38 MAPK plays an important role in responses to stress and inflammation, especially under chronic inflammatory processes. ERK is associated with cell growth and differentiation regulation. Activation of p38 MAPK via TRIF may contribute to the development of neurodegenerative diseases such as AD via increasing the β-amyloid peptides production [72,73].

Poly I:C effectively induces the maturation of dendritic cells, which enhances their ability to present antigens and activate adaptive immunity cells. Their increased expression of costimulatory molecules CD80, CD86, and cytokines such as IL-12 secretion promote naive T-cells’ differentiation into Th1-cells. It was also shown that Poly I:C can make dendritic cells shift their metabolism via increasing glucose uptake and phagocytic functions [74,75].

Thus, Poly I:C/TLR3 interaction is a complex mechanism which induces various immune and metabolic processes to secure protection from pathogens. At the same time, it could cause unfavorable pathological conditions when excessively activated. According to the literature, the injection of Poly I:C to female rodents and primates in mid-pregnancy leads to an immune response in the offspring both in the prenatal and in early postnatal periods. It is accompanied by an increase in the concentration of IL-6, IL-1β, and TNF-α in the blood serum without changing the levels of IL-4 and IL-10 [76]. This indicates that immune hypersensitivity acquired in the prenatal period in the fetus persists throughout the lifespan and is characterized by a high level of pro-inflammatory cytokine production in the early postnatal period of development without any repeated infectious stimuli.

When Poly I:C 20 mg/kg was administered repeatedly to the offspring of MIA-induced pregnant female mice, the serum concentrations of pro-inflammatory cytokines IL-6, IL-17, and TNF-α were significantly increased compared to the offspring that were not exposed to Poly I:C prenatally [77]. These changes were also accompanied by the development of postnatal liver necrosis in the offspring in a group of repeated Poly I: C administration. In addition, the mRNA expression of activating transcription factor 4 (ATF-4), a master regulator and key transcription factor of the unfolded stress response (UPR), was low in the offspring of Poly I:C-treated mice compared to the control group. The UPR is a cellular stress response triggered by the accumulation of unfolded or misfolded proteins in the endoplasmic reticulum. It is a conservative pathway aiming to restore protein homeostasis and maintain cell survival. The UPR is also able to initiate apoptosis if the stress is severe or prolonged. This phenomenon is likely caused by prenatal MIA impact [77].

A study by Zhang et al. demonstrated that the offspring of female C57BL/6J mice treated with Poly I:C on a gestation day (GD) 12.5 displayed typical features of ASD and ADHD [78]. These animals showed decreased social activity; impaired social recognition; markedly increased self-grooming, which could be interpreted as repetitive stereotypic behavior; and increased anxiety in the open field, elevated plus maze, and three-chamber tests [78]. Similar results were obtained by Zeng et al. in the adult offspring of C57BL/6J mice treated with Poly I:C on GD 12.5, who had decreased mobility, increased anxiety, frequent repetitive digging, stereotypic behavior, impaired social interaction, and impaired recognition memory, as demonstrated in the grooming test, open field, elevated plus maze, marble burying test, and three-chamber test [79]. The offspring (postnatal day (PD) 40 and 60) from Poly I:C-treated Sprague–Dawley female rats (10 mg/kg, GD9) demonstrated impairment of prepulse inhibition, which is one of the key phenotypic features of schizophrenia. There were behavioral changes associated with microglial activation in the prefrontal cortex and hippocampus, and levels of pro-inflammatory cytokines (IL-1β, IL-6, and TNF-α) varied depending on brain region and age of the animals as well [80].

The offspring (PD21) born to Poly I:C-treated CD-1 mice on GD 9.5 showed a decreased dendritic spine density in the somatosensory cortex, increased microglial synaptic pruning, and altered complement protein expression. These effects persisted in adulthood, suggesting long-term consequences of prenatal inflammation on synaptic organization and microglial activity [54]. Choi1 et al. [56] highlighted the key role of IL-17a in MIA mechanisms. They showed that Poly I:C administration induces IL-17a production by Th17 cells, resulting in microglial activation and hippocampal dysfunction in the offspring. In vivo blockade of IL-17a significantly reduced the offspring’s autistic traits, such as social withdrawal and anxiety-like behavior, highlighting its therapeutic potential [56]. Poly I:C-induced MIA resulted in an increased proBDNF level in the hippocampal CA1 and CA3 regions of the adult rat offspring. It was correlated with learning and contextual memory deficits. Infusion of anti-proBDNF antibody (p75NTR inhibitor) into the CA1 region improved neuronal synchronization and synaptic transmission as well as cognitive performance. These data demonstrate that proBDNF-mediated signaling is likely involved in MIA-induced cognitive impairment and neuronal connectivity dysfunction [81].

#### 3.2.2. MIA and LPS

LPS (Lipopolysaccharide) is a component of the Gram-negative bacteria cell wall and a TLR4 ligand. It is widely used to model MIA in animals via the induction of innate immune responses and the development of inflammation [27,28]. TLR4 is expressed on the membranes of cells implementing innate immune responses, such as dendritic cells, macrophages, monocytes, and neutrophils. TLR4 is also found in epithelial cells of the mucous membranes, endothelium, decidual cells of the placenta and beta cells of the pancreatic Langerhans islets. In the CNS, TLR4 is expressed on neurons and glial cells. It is the only member of the toll-like receptor family that activates both MyD88 (Myeloid differentiation primary response gene 88) and TRIF-dependent signaling cascade pathways. LPS binds to the cell surface protein CD14, which mediates its transfer to the TLR4/MD-2 complex [82]. This process requires the cofactor MD-2 (Myeloid Differentiation Factor 2) to stabilize LPS/TLR4 binding and mediate the conformational changes required for receptor activation [83]. In the MyD88-dependent pathway, TLR4 activation leads to the recruitment of the adaptation protein MyD88 via the TIR (Toll/IL-1 receptor) domain and initiates the formation of the Myddosome Complex [84]. As a result, the activation of interleukin-1 receptor-associated kinase (IRAK) and Transforming Growth Factor β-Activated Kinase 1 (TAK1) occurs. Its phosphorylation of IκB kinase promotes degradation of the IκB inhibitor and the release of NF-κB, as mentioned before. In the TRIF-dependent pathway, TLR4 activation occurs via the TRIF pathway, which triggers a signaling cascade including activation of TBK1 and IKKε kinases. These kinases phosphorylate IRF3, resulting in their translocation into the nucleus and induction of the interferons production similar to the TLR3 activation pathway [85].

Post-translational modifications play an important role in TLR4 activity regulation. So, the protein ubiquitination and SUMO (Small Ubiquitin-like Modifier) modification involved in TLR4 signaling pathways might both enhance or suppress the inflammatory response [86]. Metabolic changes such as oxidative stress and altered fatty acid metabolism are able to modulate TLR4 sensitivity to LPS as well [87]. The pathological consequences of excessive TLR4 activation are particularly important to discuss. Studies have demonstrated that long-term TLR4 activation might contribute to systemic inflammation development, metabolic syndrome, and the formation of pro-inflammatory background in the CNS [88,89]. TLR4-induced microglial activation was linked to the progression of neurodegenerative diseases such as AD, highlighting the importance of taking this signaling pathway into consideration [90,91].

In embryogenesis, TLR-4 is involved in the CNS development and regulates the differentiation and proliferation of NPCs [92,93]. Studies have also confirmed its role in hippocampal neurogenesis in the postnatal brain. According to the results of tests conducted in a Morris water maze, mice with TLR-4 deficiency demonstrated improved spatial memory due to increased cell proliferation and the number of mature neurons in the hippocampus. TLR4 activation mainly affects neuronal proliferation in the female brain. Inhibition of TLR-4 by TAK-242 (Resatorvid, a specific TLR4 inhibitor) increased water maze performance in young female mice compared to males and aged females [94].

MIA differently affects the offspring depending on sex. Prenatal exposure to 60 μg/kg LPS resulted in more anxious behavior in 8-week-old treated male mice compared to controls and treated females in the elevated plus maze and open field test, as well as more pronounced depressive behavior in the tail suspension test. There was also a significant decrease in the expression of granulocyte–macrophage colony-stimulating factor (GM-CSF) and *IL-12* genes in the brain cortex of treated males compared to females and higher expression of *Il1α*, *Il1β*, *Il10*, *Tnfα*, *Il12p40*, and *Il6* in treated females compared to treated males [95]. Administration of 100 μg/kg LPS to Wistar rats at GD17 led to activation of MIA, which affects the developing offspring via an increase in *Nfkb* and *App* expression level in the prefrontal cortex of both newborn males and females. Only female offspring displayed a decrease in the expression levels of the neural stem cells markers *Sox2* and *Sox9*, whereas only in males was there an increase in the expression level of *Hif1α*, which might play a neuroprotective role at this point [96].

Data are summarized in Table 2.

Rodent studies confirm that Poly I:C- and LPS-induced MIA are quite relatable for modeling and studying the mechanisms underlying the development of NPDs. The use of these MIA inducers leads to inflammatory reactions in the CNS, microglia activation, epigenetic changes, and dysregulation of neurotrophic factors such as BDNF. These findings elucidate critical checkpoints of immune-mediated adverse influence on the developing brain and suggest future research directions for the development of NPD-preventing and therapeutic strategies.

## 4. Microglia

Microglia constitute 5 to 20% of the total glial cells in the CNS and approximately 10% of all brain cells. In the adult mouse brain, their number is estimated at 3.5 million. The microglia density varies in different areas of the brain [101], with larger numbers of microglia found in the hippocampus, substantia nigra, olfactory cortex, and basal ganglia. Smaller numbers of microglia are found in the cerebellum and brainstem [102].

In early embryonic development, microglial precursors migrate from the yolk sac to the CNS and colonize the parenchyma of the developing brain via blood vessels. They differentiate into immature microglia and disperse throughout all brain regions during brain development and vascularization [103,104]. After entering the embryonic brain, their numbers increase through proliferation induced by colony-stimulating factor (CSF-1), neurotrophin-3, IL-4, and IL-5 [105]. Microglial cells account for approximately 0.5–1% of all brain cells in the embryonic period and continue to increase in the postnatal period of ontogenesis, reaching maximum density at 2 weeks after birth. During the colonization period, microglia successively distribute across all layers of the cerebral cortex and also aggregate in axonal tracts and neurogenic niches, where they support and regulate neurogenesis. The microglia colonization of the brain precedes the formation of astrocytes and oligodendrocytes [106,107,108,109,110]. Microglia promote the differentiation of neural stem cells into intermediate progenitors in the embryonic period and regulate their population size via phagocytosis [107].

### 4.1. Microglia Features and Functions

Under physiological conditions, microglia are the only immunocompetent cells in the CNS. These cells differ from macrophages and dendritic cells of the bone marrow origin by their low expression of major histocompatibility complex class II proteins (MHC) and costimulatory molecules CD40, CD80, CD86. This leads to a weakened antigen-presenting function and a limited ability to activate T cells, which prevents the development of autoimmune reactions [111]. In particular, the induction of monocyte differentiation into mature antigen-presenting dendritic cells in vitro is carried out using GM-CSF-2, IL-4, and LPS. Under similar conditions, microglia have reduced the expression of MHC II proteins and form a macrophage-like phenotype characterized by the expression of anti-inflammatory IL-10. Microglia provide protection against infectious pathogens, pathological proteins such as amyloid-beta (Aβ), aggregated α-synuclein, mutant huntingtin protein, and prions, as well as primary or metastatic CNS tumors. Microglia highly express Fc receptors, Toll-like receptors (TLRs) and viral recognition receptors to perform these functions [112].

Microglia perform high plasticity and the ability to switch between different states, such as a homeostatic, pro-inflammatory, anti-inflammatory, or pathologically activated state, depending on the processes occurring in the brain like injury, aging, neurodegeneration, etc. Neurons and astroglia control the homeostatic type of microglia, inhibiting their activation, and form an immunosuppressive environment in the CNS by expressing neuronal glycoproteins CD200, CD47, and microRNA-124-3p as well as synthesizing astroglial neurosteroids (progesterone, estradiol, dehydroepiandrosterone, testosterone) [113].

Resting homeostatic microglia are characterized by a branched structure with thin mobile processes that actively probe the microenvironment for signals of cellular injury and infectious invasion. This form of microglia helps maintain tissue homeostasis through the synthesis of neurotrophic factors such as IGF1/2, fibroblast growth factor 2 (FGF2), BDNF, and PDGF, as well as the anti-inflammatory cytokine TGF-β1 (transforming growth factor beta 1). Homeostatic microglia promote neuronal progenitor cells and oligodendrocytes survival and maturation. Pharmacological depletion of microglia in the developing brain results in increased neuronal loss and cellular debris [114]. Homeostatic microglia perform synaptic remodeling by partial phagocytosis (trogocytosis) of inactive synapses, dendritic spines, small dendrites, and excessively branched axons. Microglia predominantly eliminate excitatory glutamatergic synapses, limiting neuronal excitability and glutamate toxicity [115]. They also phagocytize apoptotic cells in the CNS, which activates the TREM2 receptor (Triggering Receptor Expressed On Myeloid Cells) stimulating phagocytosis and suppressing pro-inflammatory microglial activation [116].

It is worth noting that the molecular and functional features of microglia are subject to circadian rhythms correlated with the light cycle. According to Mattei et al., microglia undergo changes at both the transcriptomic and functional levels synchronized with light in C57BL/6J mice [117]. So, in the resting phase in rodents (day), microglia were characterized by a high level of homeostatic gene expression, including the purinergic receptor *P2ry12* and *Cd39*, mediating the conversion of extracellular ATP (or ADP) into AMP [118]. In the dark phase (the period of activity of rodents), downregulation of *P2ry12* and *Cd39* expression was observed alongside an upregulation of expression of genes associated with microglia activation and phagocytosis, such as complement *C1q* subcomponent subunit A *C1qa*, C-type lectin domain-containing 7A *Clec7a*, and *Cd11b*. Such changes indicate the transition of microglia to an activated or at least primed state. At the functional level, these molecular changes were accompanied by the dynamics of microglia morphology and movement. In the dark, microglia exhibited a more branched morphology, increased motility of processes, and active surveillance of the brain parenchyma. It is worth noting that these changes persisted even when neuronal activity was suppressed, suggesting an intrinsic circadian mechanism within microglia possibly regulated via Bmal1-dependent pathways or some systemic signals (e.g., glucocorticoids, cytokines). Dendritic spine density in the hippocampus also fluctuated throughout the day, reaching a minimum during the dark phase, which correlated with the peak of microglia activity. This might have occurred due to microglia participating in nocturnal remodeling of synaptic networks contributing to neural plasticity and learning [117]. These data highlight the active role of microglia in circadian neural network dynamics and their potential involvement in synaptic restructuring during the day. The timing of sample collection may markedly influence microglial molecular profiles in immune-mediated NPD models and is crucial when planning such studies.

**Figure 1:**
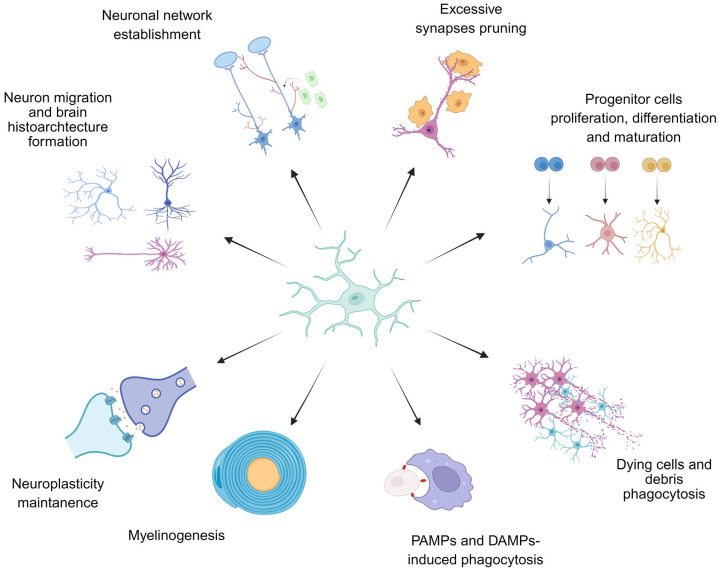
Microglia cells functions in the CNS.

The numerous functions of microglia are summarized in Figure 1.

Microglia are capable of activation depending on microenvironment signals. Pro-inflammatory activation occurs within minutes after injury or infection, causing changes in microglia genes’ expression, morphology, and functioning.

The pro-inflammatory M1 phenotype of microglia is characterized by cell body hypertrophy and short, weakly branched processes. In this state, they gain cytotoxicity and high migratory and proliferative activity to perform immunostimulation, antigen presentation, and bactericidal activity [119].

The anti-inflammatory M2 phenotype of microglia is characterized by a round (amoeboid) cell body form, enlarged size, and high phagocytic activity, as well as secretion of anti-inflammatory cytokines TGF-β and IL-10, and growth factors such as VEGFα (Vascular Endothelial Growth Factor A), PDGFA, and the neurotrophins BDNF and FGF2. This secretory shift promotes neurogenesis, synaptogenesis, angiogenesis, and mitochondriogenesis [120].

Using single-cell RNA-sequencing and transcriptome analysis technologies, specific microglia states have been identified and described in recent studies. Various phenotypes were discovered: ARM (Age-Related Microglia), DAM (Disease-Associated Microglia), MGnD (Microglial Neurodegenerative Phenotype), LDAM (Late Disease-Associated Microglia), WAM (White Matter-Associated Microglia), and DM (Dark Microglia). These states differ in terms of their functional, morphological, and molecular features, as well as localization in tissues, as they are involved in various pathological processes, including aging and neurodegeneration. The most profoundly described phenotype is DAM microglia, identified in AD [121].

DAM is a specific microglia activation state that occurs in response to neurodegenerative processes such as the accumulation of β-amyloid plaques (Aβ) or hyperphosphorylated tau protein. This phenotype is characterized by increased expression of *Trem2*, Apolipoprotein E (*ApoE*), the gene encoding Dectin-1 (C-type lectin domain family 7 member A—*Clec7a*) and lipoprotein lipase (*Lpl*), as well as decreased expression of P2Y_12_ receptor (Purinergic Receptor 12 *P2ry12*), Transmembrane Protein 119 (*Tmem119*), and the chemokine receptor (C-X3-C Motif Chemokine Receptor 1, *Cx3cr1*). Functionally, DAMs are actively involved in protein aggregates phagocytosis, lipid metabolism regulation, and pro-inflammatory reaction suppression. However, their role is controversial: in the early stages of the disease, they limit the spread of pathology, while with chronic activation they can contribute to neurodegeneration progression [122,123].

### 4.2. Sex Differences Microglia Under Physiological and Pathological Conditions

Sex differences in the incidence, prevalence, severity, and/or progression of NPDs and neurodegenerative diseases were reported in a number of studies. Thus, AD is more common in women over 65 years of age (ratio 1.6–3:1 compared to men) and is associated with more severe cognitive impairment [124,125]. PD occurs more frequently in men (ratio 3.5:1 compared to women), and women usually have slower PD progression [126,127]. Women have a lower incidence of stroke (which also depends on age), but the effectiveness of treatment after stroke in women is lower than in men [128]. Depression is more prevalent in women [129], while schizophrenia and ASD are more common in men [130,131]. Generally, with some exceptions (e.g., PD), epidemiological studies show that disorders emerging earlier in life are more common in men, whereas disorders emerging later in life are more common in women. The reasons underlying these sex differences are unknown, as they might be genetic, or related to sexual differentiation of the brain and/or circulating sex steroids. All these factors influence the activity of neurons, astrocytes, and microglia [132,133,134,135,136].

As the main immune cell of the CNS, microglia are characterized by pronounced sexual dimorphism at the molecular and functional levels, which was described in detail by Han et al. [137] These differences play a pivotal role in maintaining the homeostasis of CNS and the formation of sexual dimorphism in susceptibility to NPDs and neurodegenerative diseases’ initiation and development. The microglial phenotypes are determined by the interaction of systemic regulators, including sex hormones, as well as endogenous mechanisms associated with genotypes belonging to the male or female sex. The most pronounced changes in microglial differentiation occur in the prenatal and early postnatal periods, when the concentrations of sex hormones increase sharply. As shown in the study by Bordt et al., sex hormones, especially androgens, play a key role in microglial phenotype programming at the early stages of ontogenesis [138]. Studies have shown that the gene’s expression in microglia varies depending on the biological sex. This is consistent with the role of sex chromosome aneuploidies in the sexual dimorphism formation in psychiatric disorders [139,140].

The X-chromosome contains the largest number of immune-related genes compared to other human chromosomes, which may partially explain the more precise regulation of the immune response in females [141,142]. Estradiol, the main female sex hormone, is synthesized not only in the ovaries, but also in neurons and astrocytes. So, it interacts with estrogen receptors (ERs) of microglia in situ. This interaction reduces pro-inflammatory cytokines and ROS production and promotes anti-inflammatory activation of microglia, which plays an important role in maintaining CNS homeostasis and limiting the inflammatory response.

Analysis of microglia density in different brain regions also reveals sex differences. Guneykaya et al. demonstrated that female mice at P90 had lower microglia density in the hippocampus, cortex, and amygdala than males [143]. At the same time, Sharon et al. found no significant differences in the number of Iba1+/TMEM119+ cells in the hippocampus of age-matched rats of both sexes [144]. The presence of distinct areas with higher microglia density in males may indicate a higher density of neurons and glial cells, highlighting the complex relationship between the microenvironment and histoarchitecture.

Microglia morphology also depends on the sex. In 12-week-old rats, male microglia are characterized by larger cell bodies and short processes, whereas females have smaller cell bodies with long and branched processes [144]. In 13-week-old mice, the frontal cortex and hippocampus also show an increase in the soma size of male microglial cells compared to females, which exhibit a more branched morphology [143]. This morphology allows female microglia to scan the environment more effectively, which may contribute to a higher level of monitoring and reactivity to changing conditions.

Microglia actively participate in synaptic remodeling in the early postnatal period. A study by Prengel T.M. showed that female mice have a significantly higher synapse density in the CA1 region of the hippocampus compared to males at P14. This phenomenon correlated with increased CD68 expression by microglia, larger cell bodies, long processes, and proximity to synaptic sites. In Thy1-GFP mice of the same age, the density of presynaptic terminals is also higher in females in the same region [145]. Thus, female microglia exhibit higher levels of activity during synapse elimination and monitoring, which might be associated with more developed morphology and functional activity.

Genomic and proteomic studies confirm the presence of sex differences in microglia transcriptome and proteome. Gene Ontology analysis by Guneykaya et al. revealed that microglia from male mice exhibited increased expression of genes related to inflammatory responses, cytokine production, chemotaxis, and phagocytosis [143]. In contrast, microglia of female origin showed higher expression of genes related to cell–cell contact, migration, and the maintenance of the cytoskeleton [146]. Proteomic analysis also showed that male microglia predominantly express proteins associated with cell motility, such as Calgranulin (calcium-binding protein S100a8), as well as proteins involved in TLR-dependent signaling pathways and the detection of PAMPs and DAMPs. At the same time, in female mice, microglia displayed increased secretion of interferon-dependent proteins, indicating more pronounced antigen presentation and antiviral activity [143]. In males, the microglia of the cortex and hippocampus demonstrate a higher level of expression of MHC I and MHC II molecules compared to the microglia of females of the same age, indicating enhanced antigen presentation and participation in the adaptive immune response.

Microglia are constantly exposed to activation by PAMPs and DAMPs, so they likely contribute to the pathogenesis of NPDs due to persistent activation. Activated microglia produce pro-inflammatory cytokines and chemokines at high levels, which potentially could lead to neuronal dysfunction, loss of synaptic contacts, and an eventual decrease in cognitive functions and memory impairment.

In a study by Ince et al. [147], it was shown that microglia from old male rats (24 months) produced more of the pro-inflammatory cytokines IL-1b, IL-6, and TNF-a after ex vivo stimulation with LPS than microglia from mature male rats (12 months). In contrast, microglia from old females did not show such an inflammatory response, secreting a lower level of TNF-a protein than mature female rats’ cells, indicating a fundamental sex difference in this key neuro-immune cell population. This effect was also observed in gene expression in the hippocampus after in vivo inflammatory stimulation, which directly correlated with the pronounced behavioral changes observed in old males, but not in old females. Increased expression of both *Cx3cr1* and its cognate ligand *Cx3cl1* was detected in the hippocampus of old female rats (24 months) compared to old males. It might reflect the involvement of this pathway in the resilience of old females to hippocampal inflammation and impaired hippocampus-dependent memory formation following LPS exposure [147].

There is a critical lack of experimental data highlighting sex differences in microglia activation under different pathological conditions, particularly MIA. The most recent data are summarized in Table 3.

Sex differences in microglia exist at all levels, from morphology and density to transcriptomic and proteomic profiles. These differences depend on age, anatomical brain region, the CNS condition, and organism species. Despite significant advances in this field, the study of sex differences in microglia under physiological conditions is at a relatively early stage and requires further research to understand and develop sex-specific therapeutic approaches for NPDs and neurodegenerative diseases.

## 5. The Role of Microglia in the Development of Neuropsychiatric and Neurodegenerative Diseases

### 5.1. Autism Spectrum Disorder (ASD)

Currently, there is growing scientific evidence that ASD is closely associated with the CNS pro-inflammatory background, dysregulation of neurotransmitter levels, and neurogenesis and synaptogenesis disturbances. Particular attention is paid to pro-inflammatory events during embryonic and neonatal development such as MIA and early childhood infections, which are considered as significant triggers that affect brain development in prenatal and early postnatal ontogenesis [151]. Microglia play a critical role in the regulation of neurogenesis and determine the size of the cerebral cortex and other CNS structures, carrying out phagocytosis of NSCs and NPCs [107]. One of the key mechanisms of this phenomenon is microglia hyperactivation during intrauterine development, which is characterized by increased expression of pro-inflammatory mediators and changes in the expression of genes involved in phagocytosis. It leads to neuronal defects and negatively affects the processes of synaptic pruning and neurogenesis, resulting in structural brain changes and behavioral abnormalities in the offspring [98,152,153]. The number of microglia was increased in embryonic and neonatal mice after LPS-induced MIA, as was the number of Ki67+/Nestin+, Tbr2+ (T-box brain protein 2) NPCs in the subventricular zone. This indicates that inflammatory processes occurred in the early stages of nervous system development and abnormal activation of microglia, leading to changes in neurogenesis, impaired brain function and the emergence of ASD-like behavior in the offspring [154].

Comparison of neuropathological research data from postmortem brain samples and positron emission tomography (PET) scans of patients with ASD indicate disturbances in the morphofunctional state of microglia [155]. Microglia density was increased in the cerebral cortex and cerebellum and was accompanied by morphological changes such as enlarged cell bodies and retracted thickened processes [156,157]. Pro-inflammatory cytokines elevated levels in blood serum and cerebrospinal fluid (CSF), as well as increasing the activated microglia number of the postmortem dorsolateral prefrontal cortex, indicating that pro-inflammatory processes occur in people with ASD [158]. The expression levels of mature macrophage marker *Cd68*, pro-inflammatory cytokines *Il1b*, and anti-inflammatory *Igf1* and *Igf1r* in the gray and white matter of the anterior cingulate cortex was downregulated in males with ASD [159]. The expression of pro-inflammatory cytokines *Il18* and *Tnfa*, as well as anti-inflammatory cytokine *IL37*, was increased in the amygdala and dorsolateral prefrontal cortex in children with ASD, while the expression level of innate and adaptive immunity regulator *Il38* in the amygdala was reduced [160].

Mutations in *PTEN* gene-encoding PTEN (phosphatase and tensin homolog) protein are a risk factor for ASD. *PTEN* mutations could disrupt the complement cascade pathway in microglia, which leads to abnormal synaptic pruning during nervous system development. In mice with the *Pten m3m4/m3m4* mutation, the expression of microglial markers *Iba1* and *C1q* was significantly increased. That indirectly indicates their activated state and enhanced phagocytic capacity. The mice also displayed ASD-like behavioral patterns. Co-culture of neurons with *Pten m3m4/m3m4* microglia revealed an increased ability of these cells to achieve synaptic pruning compared to the wild type [161]. Reduced *Tmem59* expression has been observed in patients with ASD. Knockout of *Tmem59* in mice results in social deficits, repetitive behavioral patterns, and impaired communication, which are typical for ASD models. TMEM59 defects disrupt the stability of the CD93 or C1q receptor (C1qR1) in microglia, reducing their synaptic phagocytic capacity. This leads to increased excitatory neurotransmission and increased dendritic spine density [162]. Transcriptomic analysis also revealed reduced *C1q* expression in neurons in patients with Rett syndrome [163], indicating a disruption of the complement cascade in this disease. Rett syndrome is a rare neurological disorder caused by genetic mutations that disrupt brain development, leading to severe intellectual disability and almost exclusively affecting girls. It also belongs to the ASD group due to having some similar features, although it is a separate disease in terms of its clinical classification. These data support the association of ASD risk genes with the complement cascade in microglia largely depending on C1q functioning. Abnormal expression of this receptor could cause disturbances in synaptic phagocytosis by microglia, leading to pathological features such as increased dendritic spine density. Disturbances in the complement cascade, including those caused by MIA, might be one of the synapse formation and pruning disturbances that occur in people with ASD.

The absence of the postsynaptic membrane protein Neuroligin-4 (*Nlgn4*) alters microglial function and might contribute to ASD development. Differences observed between WT and *Nlgn4*−/− mice were predominantly detected in males but not in females, indicating that there is sexual dimorphism in the patterns of microglial dysfunction in *Nlgn4*−/− mice. These abnormalities include altered microglial morphology in males; a decrease in the number of process and branch points, and in the cell volume, as well as decreased antigen presentation potential, P2YR12 signaling, phagocytosis, and impaired microglial energy metabolism. Loss of NLGN4 reduced microglial surface expression of MHCI and CD54, which is associated with immune activation, in male microglial cells [148]. Sex-dependent differences in microglia presume that increased estradiol levels in females may compensate for the loss of NLGN4-related functions, providing relative compensation of microglial dysfunction. Thus, in vivo E2 supplementation was able to restore male microglial features and functions related to purinergic signaling, injury response, morphology, and annexin V binding [148]. These results are consistent with other reports of the anti-inflammatory effect of E2 on microglia in vitro and in vivo [164,165].

Accumulated research data reveal microglia cells as vital participants in ASD pathophysiological processes, especially in the context of genetic predisposition and pro-inflammatory influences. Their activation in early ontogenesis is presumably not only a hallmark, but also a driving force for disease development, which requires further study.

### 5.2. Schizophrenia

Approximately 24 million people worldwide suffer from schizophrenia, or 1 in 300 (0.32%). Among adults, this figure is 1 in 222 people (0.45%). The incidence is higher in men than in women, with an incidence ratio of approximately 1.7 (95% CI, 1.46–1.97). The etiology and pathogenesis of schizophrenia are not fully understood. It is supposed that schizophrenia may be caused by genetic predisposition and a number of environmental factors, including psychosocial ones [166,167]. Various theories have been proposed to explain the neuropathology of schizophrenia, including the dopamine, glutamate, acetylcholine, and noradrenaline hypotheses [166,168,169,170]. Over the past two decades, epidemiological studies have shown that MIA is associated with an increased risk of developing schizophrenia and other forms of mental disorders in the postnatal period. According to a meta-analysis by Khandaker GM et al., the risk of schizophrenia in the offspring exposed to MIA increases 2–5 times, with the risk of developing this disease being highest in cases of viral infections in the second trimester of pregnancy [30]. Lately, Zhou YY et al. conducted a meta-analysis of 23 independent studies, which showed a statistically significant increase in the risk of developing psychiatric disorders (odds ratio, OR = 1.25; 95% confidence interval (CI): 1.10–1.41; *p* = 0.001) in children whose mothers had infectious diseases during gestation [171].

Experimental studies revealed that MIA is associated with developmental abnormalities in key brain regions, including the cortex, hippocampus, and limbic structures [3,172], changes in which can lead to the development of psychiatric disorders. According to MRI studies, the thickness of the cortex and the volume of the hippocampus during a period of 50–100 days of postnatal ontogenesis were significantly lower in Sprague-Dawley rats exposed to poly I:C on day 15 of gestation than in animals that were not exposed to MIA [173].

Vasistha et al. showed that the offspring of 5-week-old mice showed impaired differentiation and migration of precursor cells, neuroblasts, and maturation of GABAergic interneurons in the cortex after the administration of poly I:C at a dose of 5 mg/kg on days 9.5, 12.5, and 16.5 days of gestation. The evidence allows us to suggest that it might lead to impaired formation of inhibitory neural circuits [174]. These changes are accompanied by a decrease in the expression of inhibitory transmission markers in the dorsal hippocampus, such as glutamate decarboxylase (GAD), as well as impaired functional synchronization between the dorsal hippocampus and the medial prefrontal cortex, a critically important neural circuit involved in cognitive and emotional regulation [175].

Data obtained from non-human primates, as in the case of laboratory rodents, indicate that MIA frequently leads to developmental abnormalities in the prefrontal cortex, suggesting that these effects are likely evolutionarily conserved and applicable to the human brain [176].

Furthermore, similarly to patients with schizophrenia [97], in the dopaminergic regions of the midbrain of PND 120 offspring of mice exposed to poly I:C on GD17, there is increased expression of pro-inflammatory cytokines and acute phase markers of inflammation, such as *Serpina3*, *Tnfα*, and *Il1β* [97]. This highlights the similarity of pathophysiological mechanisms in different mammalian species, including neuroinflammation and microglial dysfunction, which may determine the development of dopaminergic transmission disorders and schizophrenia symptoms.

Accumulated epidemiological data and the results of numerous studies of MIA models in animals indicate that MIA is a significant risk factor for the development of mental disorders in offspring, including schizophrenia, and requires further study.

### 5.3. Major Depressive Disorder (MDD)

MDD is a complex NPD characterized by persistent low mood, anhedonia, and loss of interest in previously enjoyable activities, and in severe cases can lead to suicidal behavior [177]. Some patients also experience somatic changes such as weight loss, fatigue, and decreased appetite. It is one of the most common mental disorders, affecting more than 250 million people worldwide [178], making it a leading cause of disability, the prevalence of which continues to rise [179]. Women are twice as likely to develop MDD compared to men [180].

A large number of studies show that abnormalities in the morphology and function of microglia in the developing brain are associated with the development of depressive disorders [181,182,183]. It was shown that abnormalities in microglia function in various brain regions, including the prefrontal cortex, anterior cingulate cortex, anterior cingulate cortex, and amygdala may play an important role in depression development [184,185,186,187,188]. During severe MDD episodes, microglia activation is observed predominantly in the anterior cingulate gyrus and anterior cingulate cortex, as determined by immunohistochemical postmortem comparative analysis of the brains of patients with and without MDD [182]. According to a comparative PET scan study of patients with MDD and healthy individuals, microglia activation in the prefrontal cortex also correlates positively with the severity of depressive episodes [189]. In a study by Liu et al. chronic unpredictable mild stress resulted in upregulated *Tnfα* mRNA level with downregulated *Arg1* and *Il10* in hippocampal microglia in female mice and upregulated *iNos* and *Il1β* with less pronounced downregulation of anti-inflammatory cytokines mRNA expression in males [150]. This discovery suggested that females were inclined to be more pro-inflammatory after stress. Afterwards, the expression of *Bdnf* and its receptor *Trkb* in the hippocampus showed a greater decrease in female compared to male mice when these animals were faced with stress stimulations. It is likely that the imbalance of microglial pro- and anti-inflammatory states as well as the BDNF-TrkB-dependent pathway in the hippocampus is involved in depressive-like behaviors. So, “microglia–neuroinflammation–BDNF” interconnection might be a fundamental mechanism contributing to sex differences in depression [150].

However, data regarding the correlation between MIA and the likelihood of developing MDD and anxiety disorders in offspring are scarce. A meta-analysis of five studies conducted on animal models with MIA showed that offspring exposed to intrauterine LPS or poly I:C spent less time in the open arms of the elevated plus maze than animals in the control groups, indicating higher levels of anxiety [190]. In a study by Sheng et al. [191], MIA was modeled by administering the TLR7/8 agonist resiquimod (RQ) to pregnant mice on GD 12.5. The results of behavioral tests showed that young (P0-P42) and adult (P52) males and females exposed to MIA during the prenatal period demonstrated less social behavior in the 3-Chamber Social Interaction Test. In the Sucrose Preference Test, the offspring of mice with MIA exhibited anhedonia-like behavior, which was more pronounced in females [191].

Although there is growing evidence pointing to the role of microglia and neuroinflammation in the pathogenesis of major depressive disorder, the link between maternal immune activation (MIA) and the development of depressive and anxiety phenotypes in offspring remains understudied: existing studies, despite demonstrating behavioral changes such as anxiety and anhedonia, are limited in number and scale, highlighting the need for larger, standardized studies to establish the mechanisms linking MIA and MDD.

### 5.4. Parkinson’s Disease (PD)

PD is the second most common neurodegenerative disease after AD. Its clinical hallmark feature is motor dysfunction manifested by bradykinesia, rigidity, balance disorder, tremor, as well as non-motor symptoms including constipation, hyposmia, depression, cognitive decline, and sleep disturbance [192]. The central pathological process in PD is the abnormal accumulation of α-synuclein protein (α-syn), which undergoes misfolding and forms aggregates called Lewy bodies. These aggregates play a key role in the initiation and exacerbation of pro-inflammatory reactions in the CNS, impaired lysosomal and vesicular transport, synaptic transport and neurotransmitter release dysfunction, and mitochondrial dysfunction development [193]. These processes contribute to accelerated disintegration of the nigrostriatal dopaminergic pathway, neurodegeneration in the substantia nigra with striatal dopaminergic denervation, and impact on other motor association circuits, leading to the diverse symptoms observed in PD patients [194]. Microglia recognize α-syn aggregates via a number of receptors, including TLRs, FcγRs, Receptor for Advanced Glycation End Products (RAGEs), scavenger receptors, and TREM2, which may contribute to their activation as well as further dissemination of α-syn throughout the CNS. The spread of α-syn among cells could be carried out via tunneling nanotubes—actin-containing cytoplasmic channels providing direct exchange between neighboring cells. At the same time, microglia can transport α-syn through exosomes, facilitating the systemic spread of pathology through neural networks. Subsequently, microglia shift to the pro-inflammatory phenotype, which activates processes such as the formation of NLRP3 inflammasomes in the cytosol and the UPR in the substantia nigra. Microglia are able to play a protective role by phagocytizing α-syn aggregates at an early stage, but as PD progresses, the ability of microglia to phagocytose it becomes impaired due to prolonged activation, pathological conformations of α-syn, and aging itself [195].

### 5.5. Alzheimer’s Disease (AD)

During AD initiation and development, microglia demonstrate both neuroprotective and neurotoxic features depending on the stage of the disease and environmental factors such as Aβ plaques’ and tau protein aggregates’ presence, distribution, number, and size, as well as the microenvironment, and the content of pro-inflammatory cytokines like IL-6, TNF, etc. [112]. In the early stages of AD pathogenesis, activated microglia primarily implement protective mechanisms, including phagocytosis of Aβ plaques, release of proteases (insulin-degrading enzyme IDE, neprilysin, MMP-9, and plasminogen), and absorption of debris [196], thus forming a physical barrier that prevents the spread of amyloid plaques [197]. In later stages of the disease, impaired Aβ clearance and tau accumulation lead to microglial dysfunction, reducing their protective functioning. The increase in the amyloid plaques’ number and size, alongside aging itself, lead to persistent microglia activation. This leads to upregulated expression and increased secretion of pro-inflammatory cytokines, mediating excessive toxic effects on astrocytes and neurons. These data highlight microglia as a potential therapeutic target in AD.

MIA is capable of promoting persistent pro-inflammatory microglia activation, which manifests in the form of upregulated pro-inflammatory gene expression and enhanced reactivity of secondary inflammatory triggers [198]. Microglia cells have a low proliferation rate compared with other hematopoietic cells and a long lifespan possibly exceeding 20 years [199]. Thus, prenatal modulation of the microglia phenotype might potentially have persistent and adverse consequences later in life. This “sensitized” or “primed” state could persist throughout the lifespan and manifest as hyperreactivity to age-related changes such as β-amyloid deposition and tau aggregate formation [12,98,200].

Another important aspect is the involvement of MIA in epigenetic dysregulation, which could be initiated by both cytokines themselves and an altered metabolic environment, including ROS. This occurs due to changes in DNA methylation, acetylation, and phosphorylation [199,200], as well as miRNA synthesis dysregulation [201]. Epigenetic changes affect genes associated with neuron survival, migration, and maturation, as well as synaptic transmission and inflammatory responses. As a result, the offspring become predisposed to the possibility of initiation and accelerated development of AD due to increased susceptibility to pathological proteins such as β-amyloid and phosphorylated tau.

MIA exposure is also associated with early and stable activation of the complement C1q–C3–CR3 pathway that is vital for physiological synaptic pruning in postnatal development. This process becomes pathologically excessive because of MIA resulting in decreased synaptic density in key brain regions responsible for cognitive functions—the hippocampus, prefrontal cortex, and amygdala. The same areas are most vulnerable in AD, in which complement reactivation and excessive microglial phagocytic activity have been observed. Early excessive synaptic pruning might potentially cause a predisposition to facilitated synaptic loss in old age, especially in the presence of phosphorylated tau fragments and β-amyloid plagues [202]. It also could occur due to insufficient neuronal network capacity, causing fewer synaptic contacts to form in the early postnatal period.

In the study by Kar et al. [100], morphofunctional and biochemical changes were assessed in the brain of the offspring of Wistar rat females after gestational exposure to LPS (intraperitoneally at a dose of 100 μg/kg at GD17). It was shown that calprotectin content was increased 4-fold in LPS-treated rats during pregnancy relative to the control group. Neuronal damage and cytoarchitecture disruption of the cortex microvascular system were observed alongside an increase in levels of APP (amyloid precursor protein) and Aβ. In newborn rats obtained from females that were administered LPS, dying neurons and an increase in the protein levels of APP and Aβ were also detected in the cerebral cortex [100]. The mRNA expression upregulation of *App* as well as *Nfkb* was observed in the prefrontal cortex of newborn male and female Wistar rats that were prenatally exposed to 100 μg/kg LPS at GD17. It was accompanied by an increase in the total microglia number in the hippocampus of prenatally LPS-treated males and a decrease in the sprouting microglia in females [96].

MIA modeling and studies of aged offspring confirm the long-term consequences of pro-inflammatory prenatal exposure. CD-1 mice offspring from females treated with 25–50 mg/kg LPS at GD15–17 were investigated at the age of 1, 6, 12, 18, and 22 months [99]. They had similar behavioral abilities and Aβ42, p-tau, and GFAP protein levels at 1 and 6 months of age. Beginning at the age of 12 months, LPS-treated offspring gradually showed decreased species-typical behavior, sensorimotor ability, locomotor activity, recognition memory, spatial learning and memory, and increased anxiety, as measured by open field, elevated plus maze, object recognition, and performance in radial six-arm water maze (RAWM) tests, as well as the Aβ42, p-tau, and GFAP protein level in different hippocampal layers compared to age-matched controls [99].

**Figure 2:**
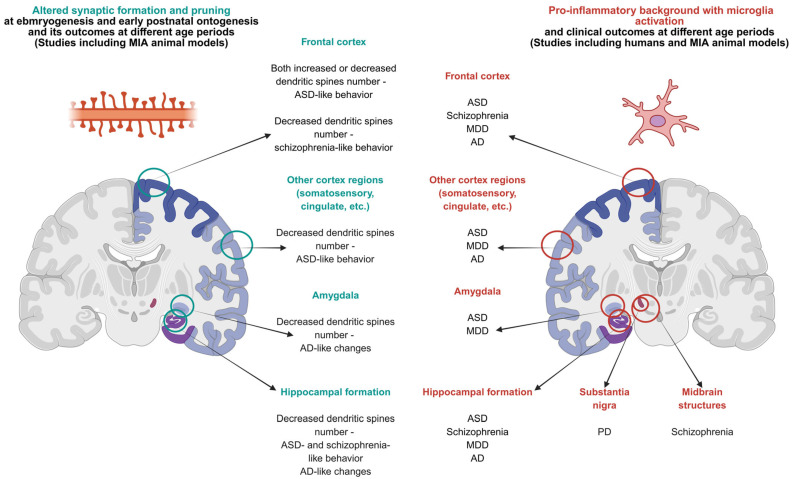
Experimental and clinical data regarding the correlation between NPDs and NPDs-like manifestations and altered synaptic pruning and pro-inflammatory background with microglia activation depending on brain regions. Most described regions with decreased dendritic spines number, likely occurred due to excessive pruning, and microglia activation and pro-inflammatory background are matched and associated with similar behavioral symptoms. Such overlaps probably indicate a causal relationship. ASD—autistic spectrum disorder; MDD—major depressive disorder; AD—Alzheimer’s disease; PD—Parkinson’s disease.

Data regarding a correlation between NPDs and NPDs-like manifestations and altered synaptic pruning and pro-inflammatory background with microglia activation depending on brain regions are summarized in Figure 2.

**Figure 3:**
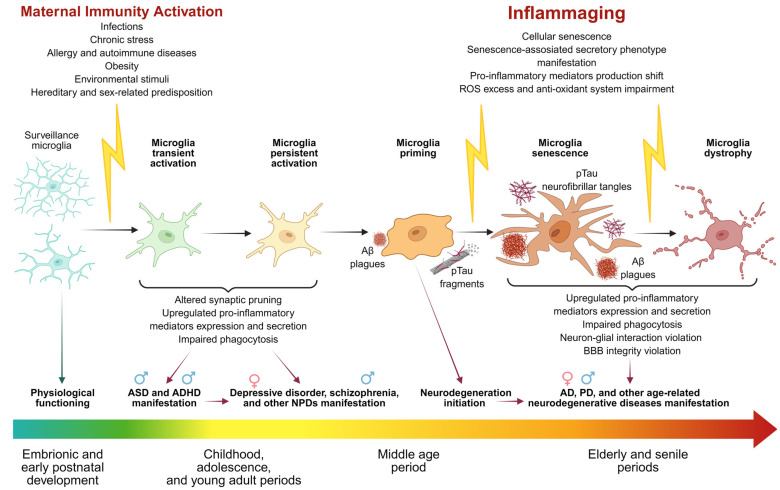
Microglia phenotypes and functioning could be changed significantly due to prenatal MIA exposure. Neuron–glia interactions could be compromised from infancy and in most cases manifest early in the form of ASD and ADHD behavior. Male microglia are more prone to pro-inflammatory responses than female ones; this allows us to suggest that the prevalence of early manifested NPDs in males could be linked to a higher and lasting pro-inflammatory background due to activation of microglia, including that which is MIA-induced. Further, persistent microglia activation might be sustained due to environmental stimuli, including psychosocial stimuli, stress conditions, metabolic disorders, and sex-related predisposition, such as X-linked genes expression and sex hormones serum level. That might explain the higher incidence and prevalence of neuropsychiatric disorders in childhood and young-adult age in men than in women. Eventually, the concentration of sex hormones, especially estradiol, decreases in women due to menopause and aging. This could contribute to more rapid exhaustion of microglia inner resources, senescence, and malfunction, leading to neurodegeneration occurring more frequently in women than in men. All these factors might be involved in sex differences in the incidence, prevalence, and severity of neuropsychiatric disorders and neurodegenerative processes throughout the lifespan (indicated in the figure). ASD—autistic spectrum disorder; ADHD—attention deficit hyperactivity disorder; NPDs—neuropsychiatric disorders; pTau—phosphorylated tau; BBB—blood–brain barrier; AD—Alzheimer’s disease; PD—Parkinson’s disease.

As mentioned before, MIA promotes long-term epigenetic and functional changes in microglia, as well as synaptic organization and inflammatory signaling pathways, potentially increasing susceptibility to various inauspicious conditions that occur later in life. Although a direct link between MIA and AD has yet to be established, a growing pool of evidence presumes that early inflammatory processes cause some imbalance of the developing CNS. These events might compromise neurocognitive stability and increase its vulnerability to neurodegenerative processes development (Figure 3).

## 6. Conclusions

The relationship between pro-inflammatory background in the CNS, microglia, and sexual dimorphism represents an important aspect of the pathogenesis of neuropsychiatric disorders and neurodegenerative diseases. Maternal immune activation caused by infections or inflammatory conditions during pregnancy plays a critical role in programming of fetal CNS development and could potentially lead to long-term changes and disturbances in the microglia functioning. These changes not only affect brain homeostasis, but can also mediate the development, causing disorders such as autism spectrum disorder, schizophrenia, Alzheimer’s disease, Parkinson’s disease, etc.

Microglia possess high-plasticity features and demonstrate significant sex differences in density, morphology, transcriptome, and functional activity. These differences could be caused by both sex hormone levels, especially that of estradiol, and genetic factors, including the expression of the genes located in sex chromosomes. In the context of MIA, these features may determine differences in vulnerability to the initiation and progression of neurological diseases. Male brains might be more susceptible to pro-inflammatory stimuli and oxidative stress, which is associated with a higher incidence of ASD in males. At the same time, females are potentially predisposed to autoimmune reactions and interferon-dependent forms of inflammatory reactions in the CNS. However, the higher prevalence, incidence, and severity of age-related neurodegenerative diseases, such as AD, in women might be associated with decreased sex hormone levels due to menopause.

In addition, increasing amounts of data implicate microglia sexual dimorphism in the development and progression of neurodegenerative diseases. So, sex differences in microglial activation may influence the efficiency of amyloid plaque phagocytosis, inflammatory signaling activation, and interactions with neurons in AD patients. This highlights the need to consider sex when developing new therapeutic strategies aimed at modulating microglial activity for more efficient AD therapy.

Understanding the complex relationships among MIA, microglia sex differences, and their functional distinctions in various pathological conditions elucidates new perspectives for personalized therapy approaches for neuropsychiatric disorders and neurodegenerative diseases. Each patient’s sex and immune status, including anamnesis vitae data regarding the mother’s pregnancy complications, should be taken into account. Further research should be focused on identifying the mechanisms underlying these differences, as well as on the application of this knowledge in clinical practice to improve the efficiency of diagnosis and therapy of neuropsychiatric disorders and neurodegenerative diseases.

## Figures and Tables

**Table 1:** MIA influence on the offspring observed in epidemiological studies.

Impact	Type of Study	Effect Size	Population	Reference
1957 influenza A2 epidemic.	Retrospective cohort study	The risk of schizophrenia in offspring was doubled in those who were in their second trimester of pregnancy during the influenza A2 epidemic in Finland in 1957	Children who were in utero during the 1957 influenza A2 epidemic in Finland	[15]
Signs of threatened miscarriage, premature birth, respiratory viral infections, moderate and severe illnesses, prenatal exposure to alcohol and nicotine, neonatal seizures, asphyxia, severe illnesses during pregnancy	Epidemiological, case–control	ADHD with a variety of factors (odds ratio, OR ≈ 2)	486 children (200 with ADHD and 286 healthy), aged 6–11 years	[34]
Genitourinary infections and preeclampsia in the mother	Retrospective cohort study	Genitourinary infections in the mother were significantly associated with an increased risk of ADHD in the child (odds ratio, OR ≈ 1.3). Preeclampsia was also an independent risk factor for ADHD (OR ≈ 1.4)	More than 84,000 children born between 1996 and 2002; children diagnosed with ADHD between the ages of 5 and 11. Medicaid billing data for pregnant women and their children in South Carolina	[35]
Family socioeconomic status (SES) and risk of developing Tourette’s syndrome (TS) and chronic tic disorder (CTD)	Prospective cohort study	Children from families with low SES had a 2–3 times higher risk of developing TS or CTD	More than 6800 children observed from birth to adolescence. UK, from the Avon Longitudinal Study of Parents and Children	[49]
Autoimmune diseases in mothers	Population-based, nested case–control	Risk of ADHD Maternal disease Multiple sclerosis (OR)1.8 95% CI 1.2–2.5 Rheumatoid arthritis(OR)1.7 95% CI 1.5–1.9 Type 1 diabetes mellitus (OR)1.6 95% CI 1.3–2.0 Bronchial asthma (OR)1.5 95% CI 1.4–1.6 Hypothyroidism (OR)1.2 95% CI 1–1.4	Children treated for ADHD in 2004–2012 (*n* = 47,944); control group—other children (*n* = 2,274,713), 1967–2008, Norwegian national registries	[41]
Autoimmune diseases in mothers	Population cohort study	Children whose mothers had any autoimmune disease had a 30% higher risk of developing CD (hazard ratio HR ≈ 1.3). rheumatoid arthritis (HR ≈ 1.6), systemic lupus erythematosus (HR ≈ 2.0)	More than 2.1 million children born in Denmark between 1978 and 2007.	[38]
Viral, bacterial, TORCH infections	Meta-analysis	Overall relative risk of developing ASD (RR): 1.13 (95% CI: 1.03–1.24), Bacterial infections were associated with a higher risk of developing ASD (RR = 1.19) than viral infections (RR = 1.09).	15 studies with a total of more than 40,000 cases of ASD (PubMed, Embase, Web of Science (before January 2016))	[23]
The mother’s body mass index (BMI) before pregnancy	Meta-analysis	Maternal overweight was associated with a moderately increased risk of ASD in the child: Relative risk (RR) = 1.28 (95% CI: 1.16–1.41), Maternal obesity increased the risk: RR = 1.36 (95% CI: 1.08–1.71)	6 cohort studies (5 prospective and 1 retrospective) and 1 case–control study. There were 8403 cases and 509,167 participants. While 5 studies were conducted in the US, 2 studies were conducted in Europe (Norway and Sweden)	[48]
Socioeconomic disadvantage (SED) and ADHD	Systematic review	35 out of 42 studies—children from families with low SES were, on average, 1.85–2.21 times more likely to have ADHD than children from families with high SES	42 studies; studies with valid ADHD diagnosis and SES measurement (income, education, occupation, marital status)	[50]
Autoimmune diseases in the mother	A population-based, multigenerational, family cohort study	Patients with OCD and SAD/HTR had high comorbidity with AID: 43% and 36%, respectively. The risk of AID was increased in first-degree relatives (parents, siblings) of patients with OCD and SAD/HTR	7,465,455 individuals born between 1940 and 2007; Swedish National Patient Register; 30,082 cases of obsessive–compulsive disorder (OCD) and 7292 cases of TD/CTD	[37]
Prenatal: Maternal depression, Gestational diabetes, Hypertension Perinatal: Cesarean section, Premature birth, Low birth weight Neonatal: Neonatal infection, Seizures in the neonatal period; hypoxia	Retrospective cohort study	Factors reliably associated with an increased risk of ASD: Prenatal: Maternal depression (OR ≈ 1.4) Gestational diabetes (OR ≈ 1.2) Hypertension (OR ≈ 1.3) Perinatal: Cesarean section (OR ≈ 1.2) Premature birth (OR ≈ 1.5) Low birth weight (OR ≈ 1.3) Neonatal: Neonatal infection (OR ≈ 1.6) Seizures in the neonatal period (OR ≈ 1.8) Hypoxia (OR ≈ 1.4)	8760 children with ASD and more than 26,280 children without ASD (control group), medical records from the US Department of Defense health care system	[43]
Validity of proposed environmental risk factors and biomarkers for autism spectrum disorder (ASD).	Umbrella review of 46 meta-analyses	Maternal overweight RR = 1.28 95% CI 1.19–1.36. Use of selective serotonin reuptake inhibitors (SSRIs) during pregnancy OR = 1.84 95% CI 1.60–2.11	67 environmental factors (544,212 cases of ASD; 81,708,787 participants). 52 biomarkers (15,614 cases; 15,417 controls). Sources: PubMed, Embase, and Cochrane until October 17, 2018	[46]
Asthma risk ADHD	Population cohort study	Asthma in mother, HR 1.41 95% CI 36–1.46 Asthma in father, HR 1.13 95% CI 1.08–1.18	Danish national registries, 961,202 live-born singleton children (1997–2012). Follow-up period: until 2016 or until ADHD diagnosis	[44]
Asthma	Population-based and family nested case–control study	Maternal asthma was associated with an increased risk of ADHD in children: OR = 1.43 (95% CI: 1.38–1.49). Asthma in the father was also associated with ASD, but to a lesser extent: OR = 1.17 (95% CI: 1.11–1.23)	1,579,263 children born in Sweden between 1992 and 2007, 22,894 cases of ASD and 228,940 controls, as well as relatives with varying degrees of kinship (full and half siblings, cousins)	[42]
Association between maternal prenatal stress and risk of (ASD)and (ADHD) in offspring	Systematic review and meta-analysis	ADHD: combined odds ratio (OR) = 1.64 (95% CI: 1.15–2.34), heterogeneity I^2^ = 90%. ADHD: OR = 1.72 (95% CI: 1.27–2.34), I^2^ = 85%	15 studies on ASD, 12 studies on ADHD. PubMed, PsycINFO, Web of Science, EMBASE, SCOPUS	[51]
Overweight/obesity before pregnancy—risk of developing ADHD	Systematic review, meta-analysis	ADHD Meta-analysis: Overweight: RR = 1.31 (95% CI: 1.25–1.38) Obesity: RR = 1.92 (95% CI: 1.84–2.00), Cohort study: Unadjusted models: HR (overweight) = 1.30 HR (obesity) = 1.92 Adjusted models: HR (overweight) = 1.21 HR (obese) = 1.60	8 cohort studies covering 784,804 mother–child pairs; 971,501 children born in Sweden between 1992 and 2004	[47]
Autoimmune diseases in the mother—risk of developing ADHD	Cohort	Cohort analysis: Any autoimmune disease: HR = 1.30 (95% CI: 1.15–1.46) Type 1 diabetes mellitus: HR = 2.23 (95% CI: 1.66–3.00) Psoriasis: HR = 1.66 (95% CI: 1.02–2.70) Rheumatic fever/carditis: HR = 1.75 (95% CI: 1.06–2.89) Meta-analysis: Any autoimmune disease: HR = 1.20 (95% CI: 1.03–1.38) Type 1 diabetes mellitus: HR = 1.53 (95% CI: 1.27–1.85) Hyperthyroidism: HR = 1.15 (95% CI: 1.06–1.26) Psoriasis: HR = 1.31 (95% CI: 1.10–1.56)	Cohort study: 63,050 children born in New South Wales (Australia) between 2000 and 2010. Meta-analysis: 5 studies included, including the current one	[40]

**Table 2:** Key alterations in the CNS due to experimental MIA depending on sex and age.

Species	Sex and Age	MIA Inducer	Dose and Gestational Period of the Inducer	The Primal Target Receptor of the Inducer	Outcomes in MIA Offspring Relative to Control	Reference
CD-1 mice	Male offspring, 2, 3, 6, 8, and 12 weeks old	PolyI:C	20 mg/kg IP, 9.5 GD	TLR3	Decreased cortical dendritic spine density since 3 w.o. Increased phagocytosis activity and microglia-mediated synaptic pruning in the somatosensory cortex and potential shifts in the developmental pattern of peak pruning from 3 to 6 w.o. Decreased PSD95 and increased C1 and C4 complement proteins’ concentration at different ages	[54]
C57BL/6J mice	Offspring sex not specified; 4 weeks old	PolyI:C	20 mg/kg IP, 12.5 GD	TLR3	Higher level of anxiety, decline in social novelty behavior, and increased self-grooming frequency as a possible repetitive stereotypic behavior Reduced microglia branches number in prefrontal cortex Increased number of dendritic spines in prefrontal cortex of the postsynaptic dense zone Increased NKCC1 protein content in prefrontal cortex	[78]
C57BL6/N mice	Male and female offspring, PND120	PolyI:C	5 mg/kg IV, 17 GD	TLR3	Upregulation of *Serpina3*, *Tnfα*, and *Il1β* in both females’ and males’ midbrain structures corresponds to similar changes in patients with schizophrenia Upregulation of *Aif1*, *Gfap*, and *Tspo* in groups with more pronounced pro-inflammatory background in both females’ and males’ midbrain structures corresponds to similar changes in patients with schizophrenia	[97]
Sprague–Dawley rats	Male offspring, 6 and 8 weeks old	PolyI:C	10 mg/kg IV, 9 GD	TLR3	Prepulse inhibition defect as a schizophrenia-like symptom at 8 w.o. Increased IBA-1+ cells in prefrontal cortex and the hippocampus, round cell bodies, and reduced arborization of microglia cells Hypertrophy and increased number of GFAP+ cells in prefrontal cortex and the hippocampus at 8 w.o. Increased IL-1β, IL-6, and TNF-a protein content in prefrontal cortex and the hippocampus at 6 and 8 w.o.	[80]
Sprague-Dawley rats	Offspring sex not specified; 8 weeks old	PolyI:C	4 mg/kg IV, 15 GD	TLR3	Increased proBDNF and decreased mBDNF protein content in CA1 and CA3 hippocampal regions; Deficit of fear-based learning; Weakened strength of phase synchronization and neural information flow in CA1 and CA3; Depressed excitatory postsynaptic currents in CA1 pyramidal neurons	[81]
C57BL/6J mice	Male and female offspring; PND0 and 8 weeks old	LPS	60 μg/kg IP, 12.5 GD	TLR4	Anxious and depressive behavior in males; Decreased GM-CSF and IL-12 content in male cortex; Upregulation of *Il1α*, *Il1β*, *Il10*, *Tnfα*, *Il12p40*, *Il6*, and *Pdl1* in female cortex	[95]
C57BL/6J mice	Male and female offspring, E15.5, PND1, PND4, and PND21	LPS	50 μg/kg IP, 12.5 GD	TLR4	Increased number of IBA-1+ cells in the hippocampus, thalamus, basal ganglia, and cortex alongside enlarged cell bodies and retracted processes (E15.5) Decrease in CD11b+/CD45+/CD86+/CD206− simultaneously with increase in CD11b+/CD45+/CD86+/CD206+ cells (E15.5 and PND4) Increased number of Nestin+ and Tbr2+ NPCs in subgranular zone of the hippocampus (PND1) Decreased number of Parvalbumin+ neurons, which corresponds to data from ASD patients, and increased number of Reelin+ neurons in males (PND21)	[98]
CD-1 mice	Male and female offspring; 1, 6, 12, 18, and 22 months old	LPS	25 or 50 μg/kg IP, 15-17 GD	TLR4	Higher number of errors and latency indicators in 12-, 18-, and 22-month-old females (LPS 50) in the radial six-arm water maze Shorter balance time in 18-month-old males and females (LPS 50) in beam-walking task Shorter time spent in open arms in 18- and 22-month-old males and females (LPS 50) in elevated plus maze Lower scores in 18- and 22-month-old males and females (LPS 25 and 50) in object location recognition task Increased levels of Aβ1-42, p-tau, and GFAP in 12-, 18-, and 22-month-old males and females (LPS 25 and 50) in different hippocampal regions	[99]
Wistar rats	Offspring sex not specified; PND0	LPS	100 μg/kg IP, 17 GD	TLR4	Increased APP, Aβ1-42, β-secretase, and γ-secretase protein content in the hippocampus and cortex Decreased BDNF protein content in the hippocampus and cortex Upregulation of *App* expression in the hippocampus and cortex Large number of TUNEL-positive cells in the cortex	[100]
Wistar rats	Male and female offspring; PND0	LPS	100 μg/kg IP, 17 GD	TLR4	Upregulation of *Nfkb* and *App* in both males’ and females’ prefrontal cortex Downregulation of *Sox2* and *Sox9* in females’ prefrontal cortex Upregulation of *Hif1a* in males’ prefrontal cortex Increased total microglia number in the hippocampus in males Decreased number of sprouting microglia in the hippocampus in females	[96]

IP—intraperitoneal injection; IV—intravenous injection; PND—postnatal day; E15.5—embryonic development day 15.5; NKCC1—Na-K-Cl cotransporter 1.

**Table 3:** Sex differences in microglia response under pathological conditions including MIA.

**Species**	**Age**	**Brain Region**	**Impact**	**Sex Differences in Microglia**	**Reference**
C57BL/6J mice	20 weeks	Hippocampus (CA3)	Neuroligin-4 gene knockout (*Nlgn4−/−)*	Males—increased expression of genes and proteins associated with antigen-presenting function MHC-II, CD74, purinergic receptors P2ry12 and P2rx4. Higher microglia density Females—higher expression of genes associated with interferon signaling Ifit1, Ifit3, immune regulation Cxcl10	[148]
C57BL/6J mice	7 weeks	Hippocampus (CA1 and dentate gyrus), prefrontal cortex	MIA (poly I:C + 24 h constant lighting)	Males—1055 differentially expressed (DE) transcripts vs. physiological control (590 down, 465 up). Male hippocampal transcriptome responds more strongly to MIA than female Females—very few DE; noticeable changes only with poly I:C + constant lightning combination: 83 DE (70 down, 13 up)	[149]
Sprague-Dawley rats	8 weeks	Hippocampus (CA1, CA3, and dentate gyrus)	Chronic unpredictable mild stress (CUMS)	Females—higher microglial activation: increase in the number of Iba1+ cells with amoeboid morphology. *Il1β* and *Tnfα* mRNA expression was significantly higher in females after stress than in males Males—microglial activation was less pronounced	[150]
Sprague-Dawley rats	12 weeks	Hippocampus, cerebral cortex, amygdala, striatum, cerebellum, olfactory bulb	Colony-stimulating factor 1 receptor (CSF1R) inhibitor PLX5622	Males—the initial density of microglia is higher compared to females After PLX5622 administration microglial density decreased by 40–60% Females—after PLX5622 reduction in microglial density was significantly higher than in males	[144]
Sprague-Dawley rats	24 months	Hippocampus (CA1 and dentate gyrus), prefrontal cortex	MIA (LPS)	Males—more pronounced M1-like activation phenotype; increased expression of pro-inflammatory genes in microglia *Il1b*, *Tnf*, *Ccl2*, increased markers of microglia activation Iba1, CD68 Females—increased expression of genes associated with inflammation, regulation, and repair *Il10*, *Arg1*	[146]
Wistar rats	PND1	Hippocampus (CA1, CA3, and dentate gyrus); prefrontal cortex	MIA (LPS)	Males—increase in the total number of microglial cells; increased expression of *Hif1α* (neuroprotective effect) Females—decrease in the number of ramified microglial cells Both sexes—increase in *Nfκb* in the prefrontal cortex.	[95]

PND1—postnatal day 1.

## Abbreviations

AD	Alzheimer’s disease.
ADHD	Attention deficit hyperactivity disorder.
AMPA	α-amino-3-hydroxy-5-methyl-4-isoxazolepropionic acid.
APP	Amyloid precursor protein.
ARM	Age-Related Microglia.
ASD	Autism spectrum disorder.
ATF-4	Activating transcription factor.
Aβ	Amyloid-beta.
BDNF	Brain-Derived Neurotrophic Factor.
Bmp2	Bone morphogenetic protein.
CCL2	C-C motif ligand 2.
Clec7a	C-type lectin domain family 7 member A.
CNS	Central nervous system.
CX3CR1	C-X3-C Motif Chemokine Receptor 1.
CXCL8	C-X-C Motif Chemokine Ligand 8.
DAM	Disease-Associated Microglia.
DAMP	Damage-Associated Molecular Patterns.
DM	Dark Microglia.
DOHaD	Developmental Origins of Health and Disease.
dsRNA	Double-stranded Ribonucleic acid.
ERK	Extracellular signal-regulated kinase.
ERRα	Estrogen receptor-related receptor.
FGF2	Fibroblast Growth Factor.
GD	Gestational day.
GFAP	Glial Fibrillary Acidic Protein.
GM-CSF	Granulocyte–Macrophage Colony-Stimulating Factor.
Gpx3	Glutathione peroxidase 3.
HIF-1	Hypoxia-inducible factor 1.
HPAA	Hypothalamus–pituitary–adrenal axis.
IDE	Insulin-degrading enzyme.
IGF1	Insulin-like grown factor.
IKK	Complex IκB kinase complex.
iNOS	Inducible NO synthase.
IRF3	Interferon Regulatory Factor 3.
JNK	c-Jun N-terminal kinases.
LDAM	Late Disease-Associated Microglia.
LPS	Lipopolysaccharide.
LTP	Long-term potentiation.
LTD	Long-term depression.
MAPK	Mitogen-activated protein kinase.
MCP-1	Monocyte Chemoattractant Protein 1.
MD-2	Myeloid Differentiation Factor 2).
MeCP2	Methyl-CpG-binding Protein 2.
MGnD	Microglial Neurodegenerative Phenotype.
MHC	Major histocompatibility complex.
MIA	Maternal immune activation.
MyD88	Myeloid differentiation primary response gene 88.
NF-κB	Nuclear factor-kappa B.
NLRP3 inflammasome	NOD-like receptor family, pyrin domain-containing 3 inflammasome.
NMDA	N-methyl-D-aspartate.
NPCs	Neural progenitor cells.
NR3C1	Nuclear receptor subfamily 3 group C member 1.
NSCs	Neural stem cells.
P2RY12	Purinergic Receptor 12.
p75NTR	p75 Neurotrophin Receptor.
PAMP	Pathogen-Associated Molecular Patterns.
PD	Parkinson’s disease.
PDGF	Platelet-derived growth factor.
Poly I:C	Polyinosinic–polycytidylic acid.
PPARγ	Peroxisome proliferator-activated receptor.
PTEN	Phosphatase and Tensin Homolog.
RAGE	Receptor for Advanced Glycation End Products.
ROS	Reactive oxygen species.
Sall1	Spalt-Like transcription factor 1.
STAT3	Signal Transducer and Activator of Transcription 3.
SUMO	Small Ubiquitin-like Modifier.
TAK1	Transforming Growth Factor β-Activated Kinase 1.
TBK1	TANK-binding kinase 1.
TGF-β1	Transforming growth factor beta 1.
TLR	Toll-Like Receptor.
TMEM119	Transmembrane Protein 119.
TNF-α	Tumor necrosis factor.
TORCH	Toxoplasma, Other, Rubella, Cytomegalovirus, Herpes.
TRAF	TNF receptor-associated factor.
TREM2	Triggering Receptor Expressed On Myeloid Cells.
TRIF	TIR domain-containing adapter-inducing interferon-β.
UPR	Unfolded stress response.
VEGFα	Vascular Endothelial Growth Factor A.
WAM	White Matter-Associated Microglia.
